# Enhancement of Rosmarinic Acid Production in Hairy Root Cultures of *Perovskia atriplicifolia* Benth

**DOI:** 10.3390/ijms26073187

**Published:** 2025-03-29

**Authors:** Renata Grąbkowska, Marta Krzemińska, Katarzyna Gaweda-Walerych, Anna Karolina Kiss, Kamila Pluta, Izabela Grzegorczyk-Karolak

**Affiliations:** 1 Department of Biology and Pharmaceutical Botany, Medical University of Lodz, 90-151 Lodz, Poland; renata.grabkowska@umed.lodz.pl (R.G.); marta.krzeminska@umed.lodz.pl (M.K.); kamila.pluta@student.umed.lodz.pl (K.P.); 2 Department of Neurogenetics and Functional Genomics, Mossakowski Medical Research Institute, Polish Academy of Sciences, 02–106 Warsaw, Poland; kgaweda@imdik.pan.pl; 3 Department of Pharmaceutical Biology, Medical University of Warsaw, 02-097 Warsaw, Poland; anna.kiss@wum.edu.pl

**Keywords:** gene expression, *Perovskia atriplicifolia*, polyphenolic compound, *Rhizobium rhizogenes*, rosmarinic acid metabolic pathway, *Salvia yangii*

## Abstract

This study reports the first successful establishment of *Perovskia atriplicifolia* hairy root cultures using *Rhizobium rhizogenes* and evaluates their potential for bioactive phenolic acid production, particularly rosmarinic acid (RA). Hairy roots were induced using two *R. rhizogenes* strains, A4 and ATCC 15834; transformation was confirmed by PCR analysis targeting the rol and aux genes. The A4 strain exhibited higher transformation efficiency (41.3%) than ATCC 15834 (30.2%). Eight transgenic root clones (C1–C8) were established and confirmed as transformed. The clones exhibited significant variation in biomass accumulation and phenolic acid production. RA production was most strongly correlated with *PAL*, *RAS*, and *CYP98A14* expression. Hierarchical clustering clustered the clones into three groups based on growth, metabolite content, and gene expression. Lines C1 and C2 exhibiting the highest RA, total polyphenol content, and the highest productivity were selected for further experiments. McCown Woody Plant (WP) and Schenk and Hildebrandt (SH) media demonstrated the greatest biomass accumulation, with growth indexes exceeding 13. Conversely, Gamborg (B5) medium enhanced RA content, achieving 38.3 and 40.8 mg/g dry weight (DW) for clones C1 and C2, respectively, representing a fourfold increase compared to the least favorable Murashige and Skoog (MS) medium. These findings establish *P. atriplicifolia* hairy roots as efficient systems for RA biosynthesis and can provide a basis for metabolic engineering and scale-up production of phenolic acids in medicinal plants.

## 1. Introduction

Modern society is subject to increasing pressure from civilization diseases, such as cancer, circulatory disorders, diabetes mellitus, obesity, and neurodegenerative diseases; their etiology is closely related to the negative effects of industrialization and Western lifestyles. In many cases, their pathomechanism is significantly associated with chronic oxidative stress, characterized by a disturbed balance between reactive oxygen species (ROS) generation and the body’s antioxidant capacity [1]. Healthy organisms employ several enzymatic mechanisms to minimize ROS-induced damage and protect against their excessive production. Additionally, exogenous antioxidants can be provided as dietary supplementation. Nevertheless, free radicals are continuously produced in aerobic organisms, and this must be counterbalanced by a similar rate of antioxidant consumption [2]. Many plant-derived products act as exogenous antioxidants, neutralizing free radicals and mitigating oxidative stress. Among these, polyphenol-rich products are of particular interest for preventing civilization diseases [2,3]. Rosmarinic acid (RA) is a polyphenol that has received significant attention as an antioxidant supplement and is used in various products, ranging from food preservation to cosmetics and pharmaceuticals [4].

Rosmarinic acid, an ester of caffeic acid and 3,4-dihydroxyphenyllactic acid, is one of the most abundant caffeic acid esters in the plant kingdom. First isolated from rosemary (*Rosmarinus officinalis* L.), RA has since been identified in many species, particularly in the Lamiaceae and Boraginaceae families [5]. RA exhibits numerous biological and pharmacological activities, including anti-inflammatory, antiviral, and antibacterial effects [4,6]. As a potent free radical scavenger, RA demonstrates neuroprotective, cardioprotective, and hepatoprotective properties and shows cognitive-enhancing effects by inhibiting the prolyl oligopeptidase and amyloid-*β* aggregation pathways. Long-term dietary exposure to RA has shown promise for cancer chemoprevention, with studies indicating therapeutic potential against various cancers. RA has been found to increase the expression of pro-apoptotic genes (*BNIP3*, *TNF*, and *GADD45A*), inhibit the anti-apoptotic protein BIRC5, arrest the cell cycle, and reduce metastatic potential in cancer cells [4,6,7].

Despite its broad occurrence, RA content rarely exceeds 1% of plant dry weight, with the precise value depending on inter alia growth phases, ecological zones, and climatic conditions [7]. The increasing demand for RA has highlighted the need for alternative production methods. Plant biotechnology offers promising solutions, providing a sustainable, environmentally friendly approach to producing valuable plant materials with consistent phytochemical profiles.

Sterilized and standardized plant cultures present an attractive alternative to traditional cultivation methods. In vitro culture allows consistent production irrespective of geographical or seasonal limitations; the product is also free of biological contaminants and enables efficient production within shorter timeframes than field cultivation [8,9]. One of the most promising types of in vitro plant systems is hairy root culture. This approach is characterized by high stability and rapid growth in hormone-free media, and biotechnologically optimized hairy root cultures can produce high amounts of bioactive compounds, often outperforming soil-grown plants [10,11]. Furthermore, in vitro cultures enable enhanced biosynthesis of bioactive compounds through strategies such as genetic modification, selection of high-yielding lines, and optimization of growth conditions [12,13]. Such plant cultures have been found to produce significant levels of RA, often higher than in natural plants [4,7].

The Lamiaceae are rich in RA and other polyphenolic acids and have a wide range of medicinal, culinary, and cosmetic uses [14]. However, in recent years, many natural habitats of medicinal plants have been destroyed due to their over exploration and the environmental pollution. One species whose potential remains underutilized, owing to its low availability and limited scientific research, is *Perovskia atriplicifolia* Benth.

*P. atriplicifolia*, reclassified as *Salvia yangii* B.T. Drew in 2017, is a Central Asian medicinal and aromatic plant. It is traditionally used as a folk medicinal herb in its natural range, including Pakistan, Turkmenistan, and Afghanistan [15]. The plant exhibits antibacterial activity and is used to heal wounds and as a remedy for fever [16]. In traditional Tibetan and Chinese medicine, *P. atriplicifolia* is reputed to be a powerful analgesic and parasiticide [15,16]. The main active compounds reported in this species include phenolic compounds, terpenoids, steroids, and essential oils [15,17]. Thanks to its traditional uses and its phytochemical diversity, as well as recent advancements in biotechnological approaches used to produce high-quality raw materials, *P. atriplicifolia* was selected for the present study.

The aim of this study was to establish hairy root cultures of *P. atriplicifolia* using the soil bacterium *Rhizobium rhizogenes*, and to examine the RA biosynthetic pathway in the clones by gene expression analysis. Finally, the growth medium was optimized for the selected clones to enhance RA accumulation.

## 2. Results

### 2.1. Hairy Root Establishment and Transformation Confirmation

**Figure 1:**
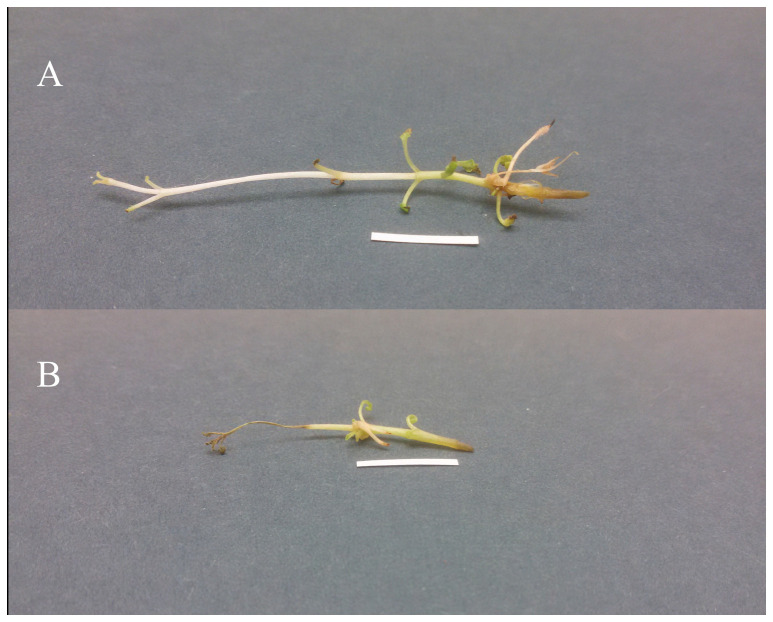
The hairy roots obtained on *P*. *atriplicifolia* shoots after infection with *R. rhizogenes* strain A4 (**A**) and strain ATCC 15,834 (**B**). Bar = 1 cm.

Hairy roots developed at the *R. rhizogenes* infection sites for both strains as a result of the plasmid DNA fragments integrating with the host plant DNA (Figure 1). However, the A4 strain demonstrated significantly superior transformation efficiency (41.3% vs. 30.2%; *p* < 0.05) (Table 1). In both cases, adventitious roots began forming at the infected sites during the first week of culture, with the process continuing for up to three weeks. The roots grew slowly and did not exceed 1 cm in length; the roots obtained from shoots infected with ATCC strain were particularly short. Roots were transferred to WP liquid medium supplemented with 500 mg/L cefotaxime and subcultured every 7 days for eight cycles to ensure complete elimination of residual *R. rhizogenes*. If the roots were not transferred to fresh WP liquid medium containing antibiotics during this period, they died.

Any roots that resumed growth in fresh medium and reached a length of 1.5–2 cm were excised from the explants during the subsequent subculture. After several subcultures, during which the bacteria used for transformation were eliminated, eight root clones (designated as C1–C8) were obtained. Notably, all the clones that were subsequently used for further experiments were initiated using the A4 strain.

**Figure 2:**
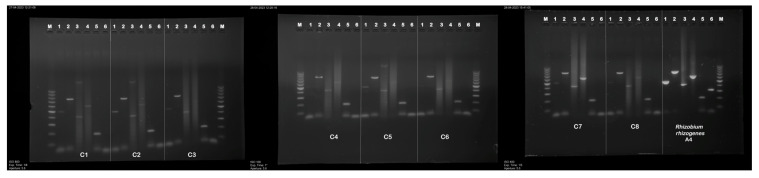
PCR amplified products in genomic DNA from the hairy root clones of *P. atriplicifolia*. Lanes: M—molecular weight marker 100–1000 bp DNA ladder, 1—*aux*1 (500 bp), 2—*aux*2 (774 bp) 3—*rol*B gene (386 bp), 4—*rol*C (582 bp), 5—*rol*D (204 bp), 6—*vir*G (319 bp).

The transgenic status of hairy roots clones (C1–C8) was confirmed by PCR analysis (Figure 2). The PCR analysis also confirmed that hairy roots were not contaminated with *R. rhizogenes*, and the *vir*G gene, which is involved in the transfer of bacterial T-DNA to the plant cell, was detected only in a positive control.

The 386 bp fragment corresponding to *rol*B, the 204 bp fragment corresponding to *rol*D, and 774 bp corresponding to the *aux*2 gene were present in all hairy root clones C1–C8 (Table 2). The band of expected size 500 bp corresponding to *aux*1 appeared in lines C1–C3, C5, and C7–C8, while the 582 bp fragment corresponding to *rol*C was identified in the clones C1–C2 and C4–C8. No amplification was observed in the negative control (genomic DNA of untransformed roots of *P. atriplicifolia*), confirming the absence of bacterial contamination and primer dimer artifacts.

### 2.2. Growth Measurement of Hairy Root Clones

**Figure 3:**
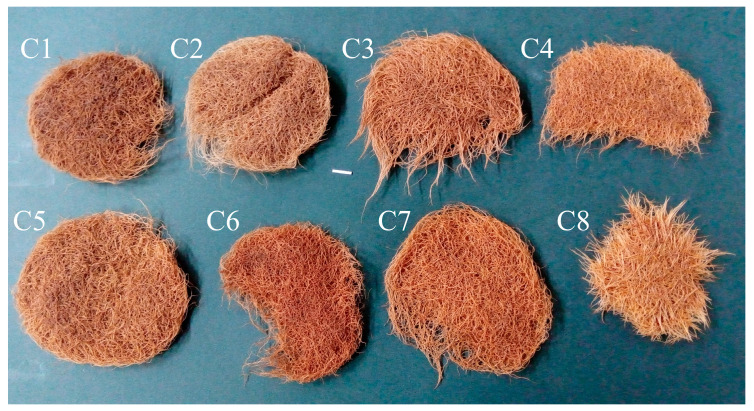
The hairy roots of *P. atriplicifolia* clone (**C1**–**C8**) grown in WP medium after 4 weeks (Bar 1 cm).

**Figure 4:**
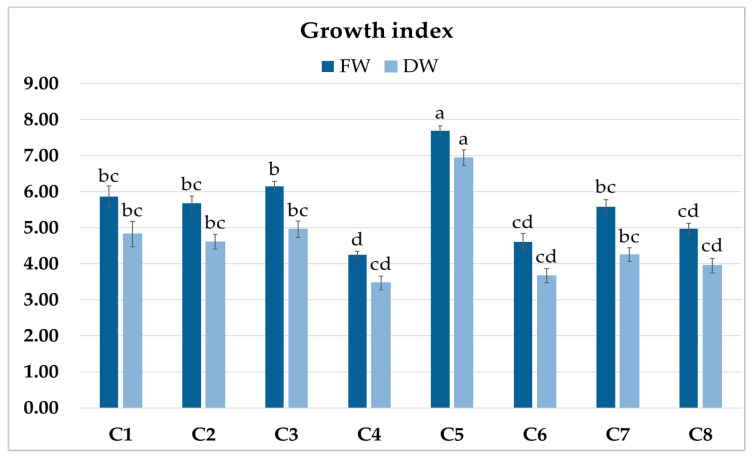
Growth index (GI) of fresh (FW) and dry weight (DW) of *P. atriplicifolia* hairy root clones (C1–C8) grown for 4 weeks in WP medium. Data represent a mean ± SEM of three independent experiments (n = 3). The results indicated by the same letter within the same parameter are not significantly different (*p* ≤ 0.05).

The roots of C1–C8 clones showed morphological features typical of hairy roots. The eight obtained root clones of *P. atriplicifolia*, confirmed as transformed, were cultured for four weeks in WP medium [18] to evaluate their growth (Figure 3). The growth index of fresh weight (FW) ranged from 4.24 to 7.68, and of dry weight (DW) from 3.42 to 6.88 (Figure 4). The highest values were achieved by clone C5, and the lowest by C4.

### 2.3. Phytochemical Studies

**Figure 5:**
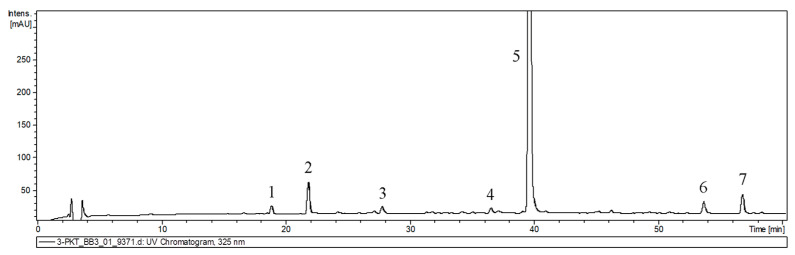
UPLC-UV chromatograms of extracts derived from hairy roots of *P. atriplicifolia* used for qualitative analysis. 1—caffeic acid dihexoside; 2—caffeic acid, 3—prolithospermic acid, 4—salvianolic acid isomer, 5—rosmarinic acid, 6—salvianolic acid F isomer I, 7—salvianolic acid F isomer II.

The qualitative analysis of methanol–water extract from hairy root cultures by UPLC-PDA-ESI-MS (ultrahigh performance liquid chromatography–electrospray ionization tandem mass spectrometry) showed that hairy roots could be suitable systems for the production of phenolic acids (Figure 5). Compounds in the extracts were identified based on retention time, as well as UV and mass spectra consistent with the literature [19,20] or by comparing their spectral properties with those observed for commercial standards. The peak characteristics and tentative identities are presented in Table 3.

The analyzed extracts were found to contain various phenolic acids. The most prevalent, rosmarinic acid, has been previously identified in the roots of this species [17]. Other compounds, including caffeic acid (CA), caffeic acid dihexoside (CDH), prolithospermic acid (PLS), salvianolic acid isomer (SAI), and salvianolic acid F (SAF) isomers, have been previously identified in other species belonging to the *Salvia* genus including their hairy root cultures [21,22,23]. The latest phylogenetic studies have placed *Perovskia*, along with other genera such as Rosmarinus, into the expanded genus *Salvia* [17].

The most abundant compound in all the obtained clones was RA (Table 4). However, its production varied in individual lines. The highest RA content was found in clone C2 (15.9 mg/g DW), with fivefold lower levels observed in the least productive lines (C4 and C7). The remaining compounds identified in the extracts were biosynthesized in hairy roots at significantly lower levels (Table 4). Although clone C2 demonstrated relatively high biosynthesis of other polyphenols, the most efficient PLS, SAI, and SAF production was observed in C1. Among the identified compounds, caffeic acid dihexoside was present in all analyzed clones, but only in trace amounts.

Finally, the highest total studied phenol (TP) content was observed in clone C2 (18.6 mg/g DW) followed by C1 (17.0 mg/g DW). The lowest level was reported for clone C4 (4.4 mg/g DW) (Table 4).

**Figure 6:**
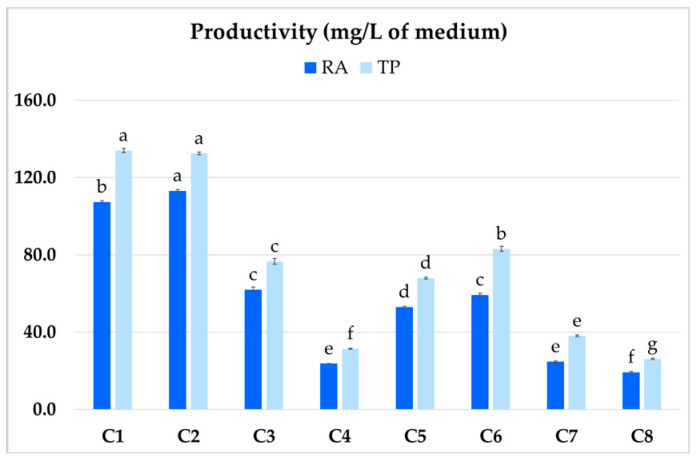
Total polyphenol (TP) and rosmarinic acid (RA) productivity (mg/L) of different clones (C1–C8) of hairy root of *P. atriplicifolia*. Data represent a mean ± SEM of three independent experiments (n = 3). The results indicated by the same letter within the same parameter are not significantly different at the level of *p* ≤ 0.05.

The highest productivity was demonstrated by clones C1 and C2 (Figure 6), which yielded 107.4 and 113.2 mg/L RA, respectively, and 134 and 132.5 mg/L TP content. The least productive clone (C4) achieved 23.7 mg/L for RA and 31.4 mg/L for TP.

### 2.4. Expression of Genes Encoding Enzymes of the RA Biosynthetic Pathway

**Figure 7:**
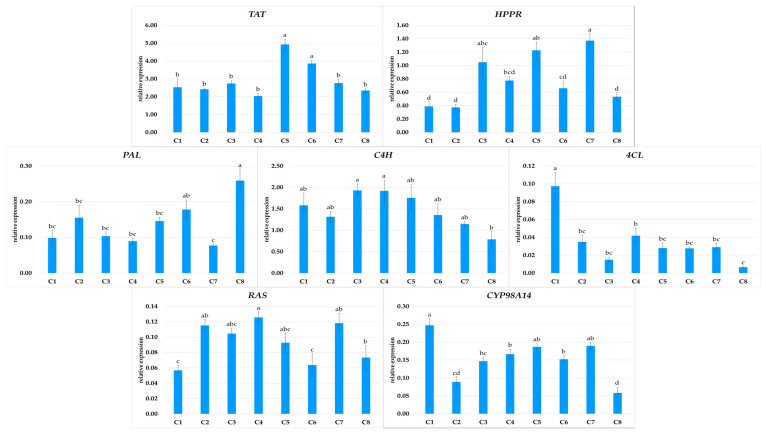
Expression of *TAT*, *HPPR*, *PAL*, *C4H*, *4CL*, *RAS*, and *CYP98A14* genes in different hairy root clones (C1–C8) of *P. atriplicifolia.* Data represent a mean ± SEM of three independent experiments (n = 3). The results indicated by the same letter within the same parameter are not significantly different at the level of *p* ≤ 0.05.

**Figure 8:**
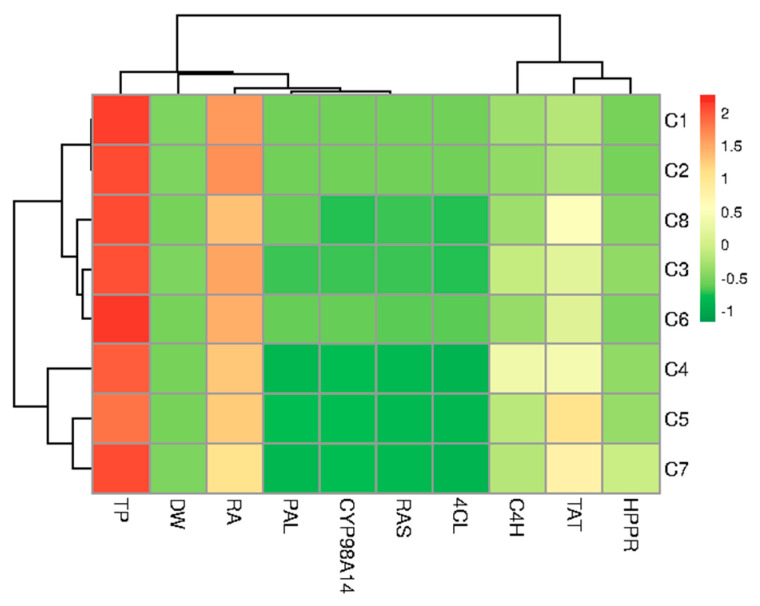
Heatmap representing the differences between hairy root clones of *P. atriplicifolia* clustered according to the analyzed parameters (growth, content of RA and TP, gene expression).

RA, the predominant metabolite in *P. atriplicifolia* hairy roots, is biosynthesized from L-tyrosine and L-phenylalanine via the phenylpropanoid pathway. In the first branch of the pathway, hydroxyphenyllactic acid is formed by TAT and HPPR; in the second branch, 4-coumaroyl-CoA is produced by reactions catalyzed by PAL, C4H, and then 4CL. These two intermediates condense in a reaction catalyzed by RAS to form 4-coumaroyl-4′-hydroxyphenyllactic acid; this intermediate is subsequently converted to RA by CYP98A14 [24]. The expression of the genes encoding all enzymes in this pathway were analyzed in the eight *P. atriplicifolia* hairy root clones, and the results were correlated with RA production (Figure 7 and Figure 8).

The expression patterns of the analyzed genes in the transformed roots were clone-specific (Figure 7). Among the genes encoding the initial steps of the pathway, *TAT* showed twice the expression in C5 as for the other clones, except for C6. *PAL* expression was at least twice as high in C8 as in the other clones; however, this clone demonstrated the lowest *C4H* and *CYP98A14* expression. The strongest expression of *C4H* and *CYP98A14*, along with *4CL*, was noted in C1.

Hierarchical clustering was used to ordinate test samples based on the correlation model into two different stimulus coordinates. From the heat map, it can be seen that the obtained clones formed three different clusters based on growth, metabolite content, and gene expression. Cluster I (C1 and C2) was characterized by high metabolite production and average growth. Cluster II (C3, C6 and C8) had moderate production and growth, while Cluster III included C5 and C7 with C4 (Figure 8).

Moreover, it was found that the production of RA in root clones of *P. atriplicifolia* is associated with the expression of *PAL*, as well as with the genes responsible for encoding the final stages of the RA pathway (*RAS* and *CYP98A14*) and to a slightly lesser extent with TP content. In addition, a good correlation was observed between the tyrosine-derived genes, *TAT* and *HPPR*. The highest RA content observed in clones C1 and C2 correlated with higher *PAL*, *CYP98A14*, *RAS*, and *4CL* expression levels but not *TAT* and *HPPR* (Figure 8).

### 2.5. Culture Condition Optimization

The two most productive hairy root clones of *P. atriplicifolia* (C1 and C2) were selected for production optimization. These were cultured in the following growth media: SH (Schenk and Hildebrandt) [25], MS (Murashige and Skoog) [26], WP (McCown Woody Plant) [18], and B5 (Gamborg) [27].

Morphological differences in the cultures were observed depending on the growth medium. In WP medium, the C1 and C2 roots were light brown, i.e., similar in color to those cultured in MS medium. However, in the MS medium, the roots were brittle and, particularly in the case of clone C2, covered with callus tissue. The presence of callus visibly restricted culture growth and complicated the transfer of roots into fresh medium.

**Figure 9:**
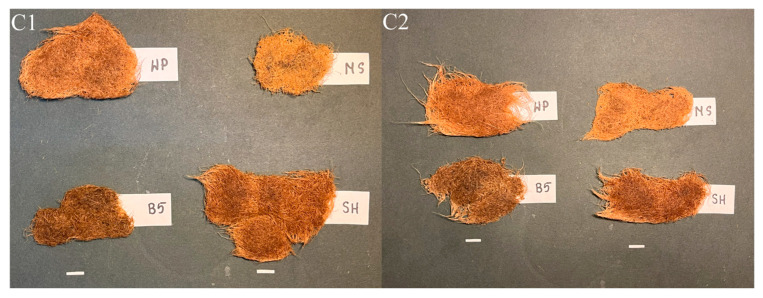
Hairy root clones of *P. atriplicifolia* (C1, C2) grown in different basal media after four weeks (Bar 1 cm). WP—Woody Plant, SH—Schenk and Hildebrandt, B5—Gamborg, and MS—Murashige and Skoog medium.

In SH medium, the C1 and C2 hairy roots exhibited a darker, orange-brown coloration (Figure 9). The cultures grown in B5 were the darkest brown in color and significantly shorter than those in WP and SH media, indicating markedly weaker growth, comparable to that achieved in MS medium.

**Figure 10:**
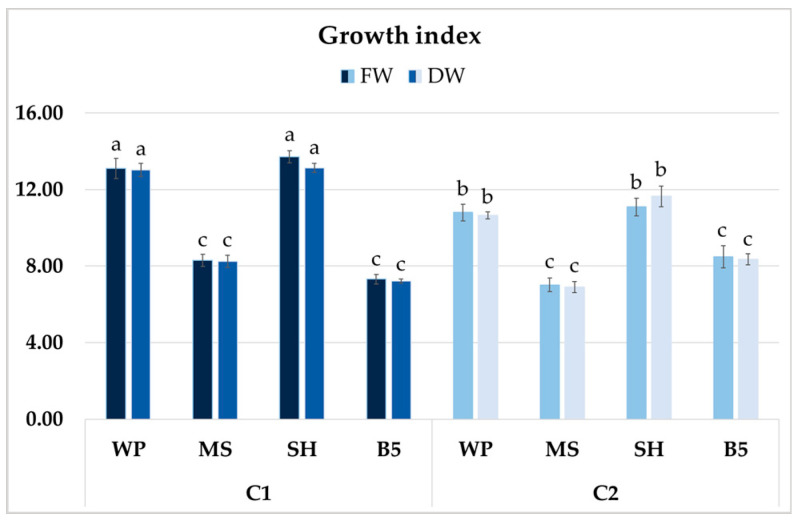
Growth index (GI) of FW and DW of *P. atriplicifolia* hairy root clones (C1 and C2) grown for 4 weeks in different basal media: WP, MS, SH, and B5. Data represent a mean ± SEM of three independent experiments (n = 3). The results indicated by the same letter within the same parameter are not significantly different at the level of *p* ≤ 0.05.

After four weeks of growth, it was determined that in some of the culture media, clone C1 exhibited higher biomass accumulation than clone C2 (Figure 10). However, no significant differences in GIs (FW or DW) were found for the clones cultured in SH and WP media; these values exceeded 13 for C1 and were approximately 11 for C2. The lowest GIs for clone C1 were recorded in B5 medium (approximately 7), a 45% reduction compared to SH medium, and for clone C2 in MS medium, with a 35–40% decrease compared to optimal conditions. The differences between clones cultivated in B5 and MS media were not significant (Figure 10).

The polyphenolic compound content was analyzed in two *P. atriplicifolia* hairy root clones collected from various basal media (Table 5).

The highest RA content was observed in both root clones cultured in B5 medium (Table 5). In these conditions, the levels of RA were similar for both clones, with no statistically significant differences: 38.31 mg/g DW for clone C1 and 40.84 mg/g DW for clone C2. These values were 3.7- to 4-fold higher than those recorded in the least favorable medium, MS. While the optimal and least favorable media for RA production were consistent for both clones, the WP medium was more favorable for RA accumulation in C1, whereas comparable RA levels were achieved in C2 cultured in SH medium (Table 5). Switching the culture medium from WP to SH for clone C1 or from SH to WP for clone C2 significantly reduced RA accumulation in the roots.

Interestingly, the biosynthesis of other polyphenols often did not correlate with RA biosynthesis. B5 medium, which was particularly beneficial for RA biosynthesis, did not stimulate the production of other metabolites, and sometimes even inhibited it (Table 5), e.g., for both tested clones, SH medium was equally or more beneficial than B5 for the biosynthesis of CA, SAF I, SAF II, and SAI.

**Figure 11:**
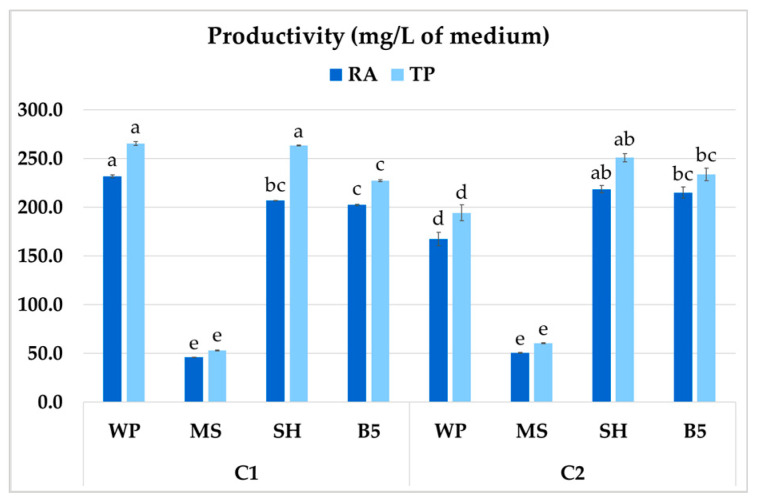
Total polyphenol (TP) and rosmarinic acid (RA) productivity of *P. atriplicifolia* hairy roots (C1, C2) cultivated in different basal media. Data represent a mean ± SEM of three independent experiments (n = 3). The results indicated by the same letter within the same parameter are not significantly different at the level of *p* ≤ 0.05.

The different medium compositions were tested with regard to their effect on culture productivity, i.e., its growth and secondary compound production (mg per liter of culture). The highest productivity of RA in *P. atriplicifolia* roots was 231.8 mg/L of medium, observed for clone C1 grown in WP medium (Figure 11). Both WP and SH medium improved total polyphenol productivity in C1 (263.4–265.6 mg/L). In contrast, for C2, both the highest RA and TP productivity were recorded in SH medium (218.7 and 251.0 mg/L, respectively). For both clones, the optimal values were as much as fivefold higher than in MS medium.

## 3. Discussion

The present study investigated various aspects of hairy root cultures in *P. atriplicifolia*, focusing on growth conditions optimization and the enhancement of RA production. Our data confirm that *Rhizobium*-mediated transformation can be performed successfully in Lamiaceae spp., as indicated by the successful establishment of hairy roots in *P. atriplicifolia* following infection with *R. rhizogenes*. Both bacterial strains used in the experiment effectively induced root formation, consistent with previous findings in other *Lamiaceae* spp. [19,28,29,30,31,32].

Strain A4 (41.3%) demonstrated higher transformation efficiency than ATCC 15,834 (30.2%), which is in line with prior reports indicating species-specific differences in transformation potential, as well as strain-specific variations in infection capability and T-DNA transfer efficiency. For instance, *R. rhizogenes* strains have exhibited varied root induction abilities in different mint species, with the highest transformation efficiency observed in *Mentha aquatic* L. infected with ATCC 15834, *M. piperita* L. and *M. longifolia* L. infected with A4, and *M. spicata* L. infected with A13 [33]. Similarly, in *Ocimum basilicum* L., strain A4 demonstrated higher infection efficiency (73%) than ATCC 15,834 (66%) [34], whereas ATCC 15,834 was more effective in *Salvia nemorosa* L. and *Salvia officinalis* L. [35,36].

To date, no reports exist on the establishment of hairy root cultures in *P. atriplicifolia*. However, successful transformation in *Perovskia abrotanoides* Karel. was noted by strain ATCC 15,834 (47.33%) [37]. These findings underscore the importance of strain selection in optimizing transformation efficiency in vitro culture systems.

*R. rhizogenes*-mediated transformation is a valuable method for enhancing bioactive compound production. The genetic background is a key determinant of phytochemical synthesis, with environmental factors playing only a secondary role [38]. To establish plants that are highly productive, it is important to screen the metabolite profiles of lines with high biosynthesis of bioactive compounds. In *Salvia virgata* Jacq. hairy root cultures, biomass accumulation ranged from 0.11 to 2.29 g, and RA content from 2.63 to 5.47 mg/g DW [39]; in contrast, in *Salvia viridis* L., hairy root clones exhibited biomass levels of 8.6–12.4 g and RA content of 10.9–35.6 mg/g DW [21]. Similar variations have been reported in other species, such as *Tylophora indica* (Burm. f.) Merrill [40] and *Verbascum xanthophoeniceum* Griseb. [41]. Line-specific differences are linked to variations in T-DNA genes, including *rol* and *aux* genes, which influence cell differentiation, auxin sensitivity, and secondary metabolism [42].

Our present findings indicate that the eight studied hairy root lines of *P. atriplicifolia* differed in regard to morphological, biomass, and metabolite content, suggesting genetic and epigenetic regulation of metabolic pathways. The obtained root lines clustered into three distinct groups, with C1 and C2 demonstrating the highest secondary metabolite content; these were selected for further research. PCR-based characterization showed the presence of *aux*1, *aux*2, *rol*B, *rol*C, and *rol*D in most lines, except C3, C4, and C6, where *aux*1 or *rol*C was absent. In the current study, no direct correlation between gene presence and growth or metabolite production was observed, because all analyzed bacterial plasmid genes were found in the genome of the two most productive clones (C1 and C2), as well as in the least productive ones (C7 and C8). On the other hand, differences among root lines with similar *rol* gene insertions could be attributed to variations in the gene copy number and integration site in the host genome [43]. Studies in *Ocimum basilicum* L. and *Melissa officinalis* L. indicate enhanced RA production in clones with multiple *rol*C copies [44]. Additionally, it is possible that the post-transformation losses of T-DNA fragments of Ri plasmids can also modulate biomass accumulation and metabolite production [45].

However, in our study, we did not analyze quantitatively the copy numbers of *aux*1, *aux*2, *rol*B, *rol*C, and *rol*D. The clones were subjected to gene expression analysis to clarify the mechanisms behind the RA biosynthetic pathway. Hierarchical clustering found RA biosynthesis correlated with *PAL*, *RAS*, and *CYP98A14* transcript levels, but not with *TAT* level (Figure 8). Our results confirm the previously reported key role of the *PAL* gene in RA synthesis. For example, *PAL* RNAi-mediated suppression in *S. miltiorrhiza* decreased RA production [46]. Other studies found correlations between RA accumulation and the activities of PAL/RAS enzymes in *Coleus blumei* Benth. cultures [47] or PAL/HPPR enzymes in *Lithospermum erythrorhizon* [48].

The relationship between RA production and gene expression from the tyrosine-derived pathway (*TAT* and *HPPR*) appears even more complex. No prominent relationship between RA accumulation and *TAT* transcripts were noted in jasmonate-induced *Prunella vulgaris* L. hairy roots [49]. Also in the present study, the ClustVis algorithm was unable to detect a correlation between RA production and *TAT*/*HPPR* levels (Figure 8). However, the algorithm focuses on static data and does not inherently account for complex dynamic changes or regulatory mechanisms, such as negative feedback that might occur in metabolite biosynthesis pathways. Nonetheless, it is worth noting that both clones of *P. artiplicifolia* with the highest RA production (C1 and C2) demonstrated low expression of the genes encoding two first enzymes of the tyrosine-derived branch (*TAT* and *HPPR*) (Figure 7). Interestingly, previous results associated high accumulation of RA with suppressed *TAT* activity in cultures of *Salvia miltiorrhiza* Bunge and *Lithospermum erythrorhizon* Siebold & Zucc. [48,50]. This invites speculation that high RA production may directly or indirectly inhibit the expression of the two initial genes of this pathway: *TAT* and *HPPR*.

Another study reported that overexpression of *TAT* and *HPPR* in *S. miltiorrhiza* hairy root cultures significantly increased RA production, suggesting their important roles in promoting RA production [51]. This apparent contradiction may be due to complex regulatory mechanisms, including potential feedback inhibition by RA or intermediates within the pathway or the transcript crosstalk phenomenon [51,52]. Regulation of metabolite levels through negative feedback mechanisms is used by plants to maintain metabolic balance and avoid overproduction of certain compounds. One example of such a regulatory mechanism within the phenylpropanoid pathway involves trans-cinnamic acid, the product of the PAL-catalyzed reaction, known to act as a feedback inhibitor of both PAL enzymatic activity and *PAL* transcripts [52,53]. The involvement of complex regulatory mechanisms at the transcriptional and post-transcriptional levels remains to be determined.

It is also worth noting that the two clones of *P. artiplicifolia* with the highest RA production are characterized by high expression of the genes encoding the two last pathway enzymes: *CYP98A14* for C1 and *RAS* for C2; this indicates molecular differences between the lines and that they can promote RA production on different stages. It could also be the reason for the slightly different results regarding the optimization of the culture medium.

Medium components, such as macro- and microelements and vitamins, play an important role in enhancing biomass and the accumulation of target compounds in culture. Therefore, four different media (MS, B5, SH, and WP) were tested to identify the optimal formulations for enhancing the productivity of the two most productive *P. atriplicifolia* root lines. In both lines, the most effective growth was observed for the WP and SH media. These findings suggest that copper may play an important role in the growth of this culture, as its concentration in WP and SH media is 8–10 times higher than in MS and B5 media. WP and SH media also contain lower amounts of nitrogen compounds, particularly ammonium ions, compared to MS medium, although the same is true for B5 medium. Moreover, WP and SH media have higher levels of pyridoxine and thiamine and lower levels of phosphate ions compared to B5 medium, which may indicate that these factors are important for the growth of *P. atriplicifolia* roots. Previous research has found that there is a reduction in phosphate levels in the culture-medium-enhanced root growth and daidzein and genistein production in cultures of *Psoralea corylifolia* L. [54].

In contrast, B5 medium was the most favorable for RA accumulation in the tested *P. atriplicifolia* hairy roots. Similarly, for *S. viridis* hairy roots, WP medium was superior for biomass accumulation, while B5 medium was comparable or superior for secondary metabolite production [21]. Also, RA level in *Salvia plebeia* R. Br. culture was greatest in B5 medium, while the highest biomass accumulation was observed in SH medium [55]. However, the RA content in *S. plebeia* roots was only 8 mg/g DW, which was approximately 4.5 times lower than that found in our *P. atriplicifolia* cultures.

On the other hand, WP and SH media provided better conditions for the production of RA and other metabolites than MS medium, which was the least suitable for both growth and metabolite accumulation. Some researchers attribute the poor growth response in MS medium to its high total nitrogen content. Nitrogen is a key factor influencing secondary metabolite production, and reduced nitrate levels have been shown to enhance biosynthesis of polyphenol in *Vitis* L. species, flavonoid in *P. corylifolia*, and RA in *Halodule pinifolia* (Miki) Hartog and *S. viridis* [54,56,57,58]. However, it has been also demonstrated that hairy roots are often less sensitive to high total nitrogen levels than to elevated ammonium concentration, which can impair cell growth by repressing nitrate assimilation. Both of these effects appear to be important for RA production in *P. atriplicifolia* cultures, where the highest RA levels were observed in B5 medium, which has the lowest ammonium-to-nitrate ion ratio; in contrast, the lowest RA accumulation occurred in MS medium, which contains the highest total nitrogen level of the tested media and the highest ammonium-to-nitrate ion ratio.

RA, which is the predominant metabolite of hairy root *P. atriplicifolia*, is also the abundant compound in such cultures of other Lamiaceae species. Highly productive hairy root cultures of *Agastache rugosa* (Fisch. & C. A. Mey.) Kuntze yielded up to 116 mg RA/g DW [59], while RA levels of 13.6 mg/g DW were reported for *Nepeta cataria* L. [60], to 35 mg/g DW for *S. viridis* [21], to 30 mg/g DW for *S. miltiorrhiza* [61], to 5 mg/g DW for *O. basilicum* [62], to 20 mg/g DW for *Dracocephalum forrestii* W. W. Smith [63], and to 16 mg/g DW for *S. nemorosa* [35].

Hairy roots could demonstrate significantly greater synthesis of secondary metabolites than wild-type roots, and metabolite production in hairy root cultures is generally more stable than in other types of cultures or in conventionally cultivated plants [21,42]. This also appears to be the case for *P. atriplicifolia*. Optimization of hairy root culture conditions increased RA levels to 40 mg/g DW, which was 20 times higher than in greenhouse-grown *P. atriplicifolia* roots (up to 2 mg/g DW) [64] and 1.5- to 2-fold higher than in field-grown plants (20–27 mg/g DW) [65]. Such large differences in the RA content in the roots of parent plants reported by two research teams may be attributed to variations in plant variety, growth conditions, or the timing of sample collection. However, they also highlight the strong sensitivity of this species to genetic and environmental factors.

Our study obtained an optimized, stable *P. atriplicifolia* transformed hairy root culture providing over 230 mg RA per liter of culture within four weeks that may be an effective alternative to field cultivation of this species. As mentioned in the introduction section, thanks to its antioxidant, anti-inflammatory, and antimicrobial properties, RA has numerous industrial applications, e.g., in pharmaceutical formulations (drugs, supplements), cosmetic skincare products, and as a natural preservative in food products [4]. Our results indicate routes of further RA biosynthesis optimization strategies for these applications, such as fine-tuning of the culture medium composition or overexpression of genes known to enhance RA biosynthesis. Additionally, optimal conditions for the metabolite production in hairy roots for industrial needs can be achieved by designing specialized automated systems for monitoring and controlling culture conditions to increase its scale, ensure consistency, and reduce labor costs.

## 4. Materials and Methods

### 4.1. Plant Material

The explants were obtained as in vitro shoot cultures of *P. atriplicifolia*. They were initiated from seeds obtained from the Direction de l’Environnement et du Cadre de Vie, Service Espaces Verts et Nature, Ville de Caen (France). The seeds were sterilized with 15% sodium hypochlorite for 20 min and rinsed three times with sterile distilled water.

For germination, the seeds were placed on MS [26] agar (0.7%) medium supplemented with kinetin (0.02 mg/L) and gibberellic acid (0.1 mg/L) and incubated in a growth chamber at 26 ± 2 °C in darkness. Following germination, the seedlings were maintained under a 16/8 h (light/dark) photoperiod, illuminated by cool-white fluorescent lamps (40 µmol/m^2^·s).

Shoot tips (0.5 cm in length) were excised from four-week-old seedlings and transferred to agar (0.7%)-solidified SH [25] medium supplemented with sucrose (3%), indole-3-acetic acid (IAA, 0.1 mg/L), and 6-benzylaminopurine (BAP, 0.5 mg/L) to obtain aseptically grown shoot culture. All growth media and growth regulators were purchased from Duchefa Biochemie B.V. (Haarlem, The Netherlands).

The plants were botanically identified by Grąbkowska based on the *Flora of Pakistan and Flora of China* [66]. A voucher specimen was deposited in the Department of Biology and Pharmaceutical Botany, Medical University of Lodz (Poland).

### 4.2. Establishment of Hairy Root Cultures

Two agropine strains of *R. rhizigenes*: ATCC (pRi15834) and A4 (pRiA4) (from LGC Standards Sp. z o.o., Kielpin, Poland), were used for the transformation. The bacteria were grown on YMB [67] solid medium at 26 °C for 48 h.

Aseptic *P. atriplicifolia* shoots, approximately 1–1.5 cm in length with 2–3 pairs of leaves, were pricked at the nodal regions with a needle immersed in a suspension of the bacterial culture. Control shoots were wounded similarly with a sterile needle without bacteria. The culture was conducted on hormone-free SH agar medium in the dark for three weeks. After this period, the frequency of transformation was evaluated as the percentage of inoculated shoots forming roots relative to the total number of shoots used for transformation. Additionally, the number and length of roots emerging from the responding explants were recorded. No roots developed in the control group.

Roots formed on infected shoots were transferred into 100 mL Erlenmeyer flasks containing 25 mL liquid WP medium [18] supplemented with cefotaxime (500 mg/L) and subcultured every 7 days for eight cycles to eliminate bacteria. The roots were incubated at 26 ± 2 °C in the dark on a rotary shaker at 70 rpm.

Eight root clones (C1–C8) exhibiting vigorous growth were selected for further cultivation.

### 4.3. Confirmation of the Transformation

The transformation process was confirmed by polymerase chain reaction (PCR). The genomic DNA from eight transformed root clones (C1–C8) and untransformed roots from 12-month-old *P. atriplicifolia* plants grown in a greenhouse (negative control group) were extracted using a Genomic Mini AX Plant Kit (A&A Biotechnology, Gdynia, Poland). During isolation, 150 mg of fresh plant materials was powdered in liquid nitrogen. The DNA sample was used as a template in PCR analysis to determine the presence of the *aux*1, *aux*2, *rol*B, *rol*C, and *rol*D genes. Additionally, PCR analysis for the *vir*G gene was performed to confirm the absence of bacteria in the hairy roots. An R_i_ plasmid isolated from 24 -hour cultures of *R. rhizogenes* strain A4 (OD600 = 0.4) using Plasmid Mini AX Kit (A&A Biotechnology, Gdynia, Poland) was used as a positive control. The sequences of primers and PCR conditions have been previously published by Skała et al. [68]. The amplification products were examined by electrophoresis, as described by Wojciechowska et al. [22]. The gels were documented using a FastGene^®^FAS-Digi PRO imaging system (Nippon Genetics Europe GmbH, Düren, Germany).

### 4.4. Hairy Root Cultivation

Eight clone hairy roots (C1–C8) were maintained in 300 mL Erlenmeyer flasks with 80 mL of WP medium and subcultured every four weeks by transferring about 0.63–0.84 g fresh weight (FW) of roots (0.08–0.13 g dry weight (DW)) into fresh medium. After 28 days, the growth of hairy roots was measured in terms of FW and DW, and the growth indexes (GIs) were calculated as described by Grzegorczyk-Karolak et al. [69]. The experiment was repeated three times (subcultures 24–26).

### 4.5. The Phytochemical Analysis

After harvesting, the plant material was lyophilized (Freeze-dryer Alpha 1–2 LD, Martin Christ, Osterode am Harz, Germany) and micronized. The plant material for each treatment (0.1 g) was extracted with 30 mL of 80% methanol in a sonication bath (Techpan, Warsaw, Poland) at 40 °C for 15 min. The extractions were repeated two more times with less solvent (10 mL). The filtered extracts were combined, evaporated to dryness, and stored in a refrigerator (4 °C) before analysis.

The qualitative analysis compounds in the extract was performed with UPLC-PDA-ESI-MS using a UPLC-3000 RS apparatus (Dionex, Germering, Germany) with DAD detection and an AmaZon SL ion trap mass spectrometer with an ESI interface (Bruker Daltonik GmbH, Bremen, Germany) and a Zorbax SB-C18 column (150 × 2.1 mm, 1.9 μm) (Agilent, Santa Clara, CA, USA). The process and details of the analysis have been described earlier [69].

All extracts were quantitatively analyzed using an Agilent Technology 1200 chromatograph with a photodiode array detector (PDA), thermostat, and autosampler (Agilent Technologies, Santa Clara, CA, USA). The analysis was performed using an Eclipse C18 XDB (4.6 × 150 mm) with a particle size of 5 μm or the equivalent column. The mobile phase (A) was 0.1% formic acid in water, and the mobile phase B was acetonitrile. The following gradient system was used for the analysis: 0 min 10% solvent B, 0–5 min 10–18% solvent B, 5–20 min 18–38% solvent B, 20–25 min 38–100% solvent B, 25–30 min 100% solvent B, 30–37 min 100–5% B (equilibration). The flow rate was 1.6 mL/min. The injection volume was arranged as 10 μL. Compounds were detected at λ = 325 nm, and their content was expressed in mg/g DW. The calibration curves for quantitative analysis were constructed based on the peak area. Authentic standards of available compounds (Chem Faces Biochemical Co., Ltd., Wuhan, China) were used for calibration. For the bioactive compounds lacking a commercial standard, quantification was based on the calibration curve of a similar compound from the same group. Total polyphenolic content was obtained as the sum of the contents of all polyphenols quantified in the extracts.

### 4.6. Gene Expression Analysis (RNA Extraction, Reverse Transcription, and Real-Time PCR (RT-PCR)

RNA was extracted with a Total RNA Midi Kit (A&A BIOTECHNOLOGY, Gdańsk, Poland) according to standard protocol. The concentration and purity of RNA were established photometrically (spectrophotometer *NanoReady* Touch, *Life Real*, Hangzhou, China), and cDNA was synthesized following the producer’s instruction using a NG dART RT cDNA synthesis kit (EURx Molecular Biology Products, Gdańsk, Poland) using random hexamer primers (PCR Thermal Cycler T 100, Bio-Rad Laboratories Inc., Hercules, CA, USA). Quantitative real-time PCR analysis was performed with qPCR-HS Mix SYBR^®^ (A&A BIOTECHNOLOGY, Gdańsk, Poland) following the producer’s instructions using a StepOne Plus system (Applied Biosystems, Foster City, CA, USA). Changes in gene expression were determined with the ∆∆Ct method using actin levels for normalization. The primer sequences for *P. atriplicifolia* have been published previously [24]. The study used primers for the key enzyme-encoding genes involved in phenolic acid biosynthesis, such as phenylalanine ammonia-lyase (PAL), cinnamic acid 4-hydroxylase (C4H), hydroxycinnamate coenzyme A ligase (4CL), tyrosine aminotransferase (TAT), 4-hydroxyphenylpyruvate reductase (HPPR), rosmarinic acid synthase (RAS), and a cytochrome P450-dependent monooxygenase (CYP98A14).

### 4.7. Optimization of Hairy Root Growth Condition

The selected hairy root lines (C1 and C2) were cultivated in different growth media—WP [18], SH [25], MS [26], and B5 [27]—to optimize growth and bioactive compound production (subculture 42–44). After four weeks, culture growth and polyphenol content were analyzed according to the methods described above.

### 4.8. Data Analysis

The experiments were conducted at least in triplicate for each treatment. The results were calculated as mean ± standard error of the mean (SEM). The outcomes of the different treatments were verified using one-way analysis of variance, followed by post hoc Tukey’s test, assuming min. α = 0.05 (Statistica13.1Pl, StatSoft, Krakow, Poland). Hierarchical clustering was performed using the ClustVis web tool after row centering, with the following settings for both row and column clustering: correlation as the distance metric and average as the clustering method [70]. A heatmap was constructed, where positive values indicate that the corresponding data points are above the mean after row centering for each parameter, while negative values indicate that they are below the mean.

## 5. Conclusions

This study provides insights into the significance genetic and environmental factors in shaping the growth and metabolic output of hairy root cultures. The superior performance of strain A4 and the differential productivity among clones highlight the potential for transformation optimization, clone selection, and the importance of tailored approaches of genetic engineering in optimizing hairy root systems to maximize metabolite biosynthesis.

Our findings confirm the feasibility of using optimized hairy root cultures of *P. atriplicifolia* for producing bioactive compounds, particularly RA. Comparative analyses with other Lamiaceae species suggest that *P. atriplicifolia* hairy roots could serve as a robust platform for the large-scale production of phenolic acids. Further studies integrating transcriptomic and proteomic analyses could elucidate the molecular mechanisms driving these variations and support the development of efficient biotechnological platforms for the production of high-value phytochemicals. Future experiments should focus on further optimization of the *Perovskia* hairy root culture system and increase its scale to allow higher productivity of RA, given its importance in the field of food, cosmetic, pharmaceutical, and nutraceutical industries.

## Figures and Tables

**Table 1:** Induction of hairy roots of *P. atriplicifolia* from shoot explants with *R. rhizogenes*.

Bacterial Strain	Number of Explants	Explants Forming Roots (%)	Mean Number of Roots/Explants	Mean Length of Roots (cm)
A4	63	41.3 ± 3.0 a	2.5 ± 0.34 a	0.77 ± 0.08 a
ATCC 15834	53	30.2 ± 1.0 b	2.5 ± 0.37 a	0.67 ± 0.10 a

Data represent a mean ± SEM of three independent experiments (n = 4). The results indicated by the same letter within the same parameter are not significantly different (*p* ≤ 0.05).

**Table 2:** Confirmation of *R. rhizogenes *plasmid integration into genomic DNA derived from hairy root clones (C1–C8) of *P. atriplicifolia*. *R. rhizogenes rol*B, *rol*C, *rol*D, *aux*1, and *aux*2 genes were amplified by PCR using the specific primers. Integration status of *R. rhizogenes* T-DNA genes in hairy root clones (C1–C8): ‘+’ indicates successful amplification; ‘−’ indicates no amplification.

Clone	*aux*1 (500 bp)	*aux*2 (774 bp)	*rol*B (386 bp)	*rol*C (582 bp)	*rol*D (204 bp)
C1	+	+	+	+	+
C2	+	+	+	+	+
C3	+	+	+	−	+
C4	−	+	+	+	+
C5	+	+	+	+	+
C6	−	+	+	+	+
C7	+	+	+	+	+
C8	+	+	+	+	+

**Table 3:** UPLC-PDA-ESI-MS data of polyphenolic compounds detected in *P. atriplicifolia* hairy roots.

Peak No.	Rt	[M-H]^−^	Main Fragments	Tentative Compound
1	18.9	503	341, 281, 251, 179	Caffeic acid dihexoside
2	21.8	179	135	Caffeic acid
3	27.9	357	313, 269, 203	Prolithospermic acid
4	36.4	717	519, 475, 365, 339	Salvianolic acid isomer
5	39.7	359	223, 197, 179, 161	Rosmarinic acid
6	53.6	313	269, 161	Salvianolic acid F isomer I
7	56.6	313	269, 203, 161	Salvianolic acid F isomer II

**Table 4:** Polyphenol content of different clones (C1–C8) of *P. atriplicifolia* hairy roots.

	C1	C2	C3	C4	C5	C6	C7	C8
CA	0.592 ± 0.005 b	0.443 ± 0.005 cd	0.333 ± 0.005 f	0.323 ± 0.003 f	0.464 ± 0.005 c	0.701 ± 0.011 a	0.414 ± 0.002 e	0.428 ± 0.003 de
PLS	0.101 ± 0.001 a	0.109 ± 0.003 a	0.057 ± 0.001 c	0.012 ± 0.001 d	0.084 ± 0.005 b	0.105 ± 0.002 a	0.078 ± 0.002 b	0.058 ± 0.003 c
SAI	0.719 ± 0.006 a	0.628 ± 0.005 c	0.335 ± 0.005 d	0.357 ± 0.003 d	0.293 ± 0.002 e	0.690 ± 0.013 b	0.296 ± 0.004 e	0.160 ± 0.002 f
RA	13.6 ± 0.093 b	15.90 ± 0.080 a	6.67 ± 0.136 d	3.31 ± 0.023 g	5.39 ± 0.041 e	8.28 ± 0.124 c	3.22 ± 0.054 g	3.73 ± 0.061 f
SAF I	0.449 ± 0.008 b	0.381 ± 0.009 c	0.161 ± 0.006 f	0.084 ± 0.002 g	0.134 ± 0.004 f	0.576 ± 0.014 a	0.278 ± 0.006 d	0.237 ± 0.002 e
SAF II	1.52 ± 0.017 a	1.15 ± 0.004 c	0.685 ± 0.012 d	0.312 ± 0.001 g	0.552 ± 0.009 e	1.25 ± 0.023 b	0.666 ± 0.004 d	0.467 ± 0.004 f
TP	17.0 ± 0.123 b	18.6 ± 0.088 a	8.24 ± 0.162 d	4.40 ± 0.026 g	6.92 ± 0.047 e	11.6 ± 0.175 c	4.94 ± 0.059 f	5.08 ± 0.072 f

CA—caffeic acid, PLS—prolithospermic acid, SAI—salvianolic acid isomer, RA—rosmarinic acid, SAF I and SAF II—salvianolic acid F I and II isomers, TP—total phenols; data represent a mean ± SEM of three independent experiments (n = 3). The results indicated by the same letter within the same parameter are not significantly different at the level of *p* ≤ 0.05.

**Table 5:** Polyphenol content of *P. atriplicifolia* (C1 and C2) hairy roots cultivated in different basal media.

Clone	C1				C2			
Medium	WP	MS	SH	B5	WP	MS	SH	B5
CA	0.839 ± 0.016 a	0.224 ± 0.000 e	0.738 ± 0.006 b	0.573 ± 0.005 c	0.640 ± 0.025 c	0.380 ± 0.005 d	0.588 ± 0.011 c	0.422 ± 0.012 d
PLS	0.142 ± 0.005 de	0.040 ± 0.002 f	0.154 ± 0.001 cd	0.212 ± 0.003 b	0.167 ± 0.003 c	0.130 ± 0.005 e	0.125 ± 0.001 e	0.257 ± 0.007 a
SAI	0.932 ± 0.010 bc	0.106 ± 0.002 f	1.01 ± 0.012 b	0.444 ± 0.006 d	0.994 ± 0.052 b	0.207 ± 0.003 e	1.28 ± 0.015 a	0.884 ± 0.028 c
RA	26.80 ± 0.183 c	9.48 ± 0.051 f	20.00 ± 0.033 e	38.31 ± 0.164 b	22.60 ± 0.933 d	11.00 ± 0.121 f	26.80 ± 0.454 c	40.84 ± 1.06 a
SAF I	0.282 ± 0.023 b	0.278 ± 0.007 b	0.804 ± 0.002 a	0.859 ± 0.015 a	0.245 ± 0.028 b	0.229 ± 0.009 b	0.273 ± 0.014 b	0.275 ± 0.032 b
SAF II	1.71 ± 0.022 b	0.807 ± 0.026 d	2.74 ± 0.009 a	2.65 ± 0.033 a	1.58 ± 0.069 b	1.24 ± 0.019 c	1.69 ± 0.041 b	1.66 ± 0.087 b
TP	30.7 ± 0.212 b	10.9 ± 0.085 d	25.4 ± 0.047 c	43.1 ± 0.223 a	26.2 ± 1.09 c	13.2 ± 0.156 d	30.8 ± 0.533 b	44.3 ± 1.21 a

CA—caffeic acid, PLS—prolithospermic acid, SAI—salvianolic acid isomer, RA—rosmarinic acid, SAF I and SAF II—salvianolic acid F I and II isomers, TP—total phenols. Data represent a mean ± SEM of three independent experiments (n = 3). The results indicated by the same letter within the same parameter are not significantly different at the level of *p* ≤ 0.05.

## Data Availability

The data are contained within the article.
